# Emerging Role of Eukaryote Ribosomes in Translational Control

**DOI:** 10.3390/ijms20051226

**Published:** 2019-03-11

**Authors:** Nicole Dalla Venezia, Anne Vincent, Virginie Marcel, Frédéric Catez, Jean-Jacques Diaz

**Affiliations:** Univ Lyon, Université Claude Bernard Lyon 1, Inserm U1052, CNRS UMR5286, Centre Léon Bérard, Centre de Recherche en Cancérologie de Lyon, 69008 Lyon, France; nicole.dalla-venezia@lyon.unicancer.fr (N.D.V.); anne.vincent@lyon.unicancer.fr (A.V.); virginie.marcel@lyon.unicancer.fr (V.M.); frederic.catez@lyon.unicancer.fr (F.C.)

**Keywords:** specialized ribosome, ribosome composition, rRNA modification, ribosomal protein, translational regulation

## Abstract

Translation is one of the final steps that regulate gene expression. The ribosome is the effector of translation through to its role in mRNA decoding and protein synthesis. Many mechanisms have been extensively described accounting for translational regulation. However it emerged only recently that ribosomes themselves could contribute to this regulation. Indeed, though it is well-known that the translational efficiency of the cell is linked to ribosome abundance, studies recently demonstrated that the composition of the ribosome could alter translation of specific mRNAs. Evidences suggest that according to the status, environment, development, or pathological conditions, cells produce different populations of ribosomes which differ in their ribosomal protein and/or RNA composition. Those observations gave rise to the concept of “specialized ribosomes”, which proposes that a unique ribosome composition determines the translational activity of this ribosome. The current review will present how technological advances have participated in the emergence of this concept, and to which extent the literature sustains this concept today.

## 1. Introduction: The Current Textbook Overview of the Translational Regulation

Eukaryotic gene expression follows many steps that are stringently controlled to adapt this expression to environmental challenges. Among these different steps, transcription and translation represent the first and last steps, respectively. While the role of transcriptional regulation in gene expression control is at present well-established and indisputable after extensive studies, that of translational regulation remains understudied and unclear. This could be due to the fact that analysis of translational regulation is relatively more complex than that of transcription. Transcription has been extensively analyzed thanks to genome-wide approaches since the development of transcriptomic analyses in the early 2000s and later on for chromatin availability and compaction. In contrast, such dedicated approaches were lacking for studying specifically translational step until recently. The impact of translational regulation on gene expression regulation was recently highlighted through several large-scale comparative proteomic and transcriptomic analyses in yeast [1], mouse [2], and human [3] that unambiguously showed that mRNA and protein levels were correlated for only half of the genes [4].

**Figure 1:**
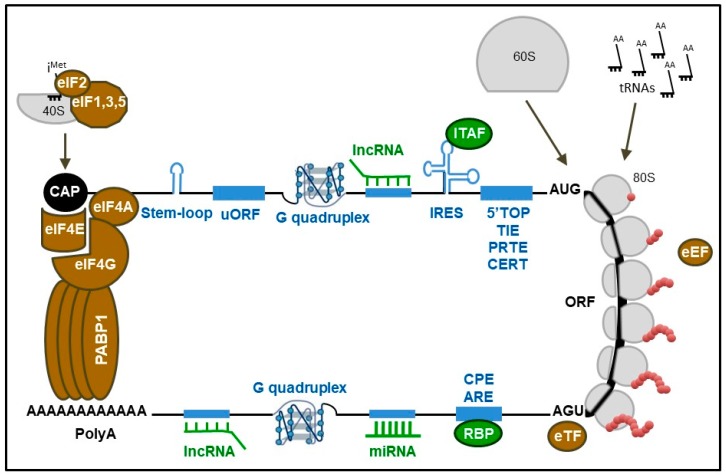
Key regulators of translation. The main effectors of the translational machinery are the ribosome (in grey) and the canonical trans-regulators (in brown) regulating the initiation, elongation and termination of translation. Additional trans-regulators of translation (in green), including RNA binding proteins (RBPs), internal ribosome entry site (IRES)-trans acting factors (ITAFs), miRNAs and lncRNAs, act in concert with the canonical trans-regulators to modulate the translational efficiency of subsets of mRNAs. These trans-regulators interact with cis-elements of the targeted mRNA, called cis-regulators (in blue), including binding sites for RBP such as cytoplasmic polyadenylation elements (CPEs) and AU-rich elements (AREs), IRES, stem-loops and G-quadruplex. In addition, several cis-regulators, including upstream open reading frames (uORFs), 5′-terminal oligopyrimidine tract (5′TOP), translation inhibitory element (TIE), pyrimidine-rich translational element (PRTE), and cytosine-enriched regulator of translation (CERT) modulate the translational efficiency of subsets of mRNAs during the initiation step. (adapted from Marcel, Oncogene, 2015, [6]).

Translation results in the synthesis of proteins corresponding to specific messages that are contained within mRNAs. The regulation of translation is dependent on cell status, environment, development, and pathological conditions [5]. Translation is regulated through a dynamic interplay between mRNA structures and/or sequences, called cis-regulators, and the translational machinery composed of its main effector, the ribosome, and trans-regulators, i.e., eukaryotic factors involved in initiation (eIF), elongation (eEF) and termination (eTF), as well as many secondary elements, a vast majority of which remain to be identified (Figure 1). For many years, ribosomes have only been considered to be the effectors of translation with no direct regulatory activity on the translational process. It was assumed that regulation of translation was exclusively performed by the cis- and trans-regulators. However, during the past decade, the notion of “specialized ribosomes” has emerged, highlighting the ribosome as an unexpected actor of translational regulation.

Translation initiation is the main rate-limiting stage of translation and comprises several steps (reviewed in References [5,7,8]). Briefly, the eukaryotic initiation factor 4F (eIF4F) complex, which consists of eIF4E, eIF4G and eIF4A, binds the 5′ end of the mRNA and interacts with the poly(A)-binding protein 1 (PABP1) leading to the pseudo-circularization of mRNA. Next, the 43S pre-initiation complex, which includes eIF3, eIF1, eIF1A, eIF2-GTP-tRNAiMet, eIF5 and the 40S ribosomal subunit also called 40S small subunit (SSU), is recruited to the 5′ cap structure (m7GpppN, where N is any nucleotide) of the mRNA, to form in association with eIF4F, the 48S pre-initiation complex which scans mRNA toward the start codon. Finally, the SSU permits the recruitment of the 60S ribosomal subunit also called 60S large subunit (LSU) which contains three tRNA binding sites called amino-acyl (A), peptidyl (P) and exit (E). Joining of both ribosomal subunits results in an active 80S ribosome with the tRNAiMet in the P site. During translation elongation, tRNA enters the ribosome at the A-site, where decoding takes place in the SSU (i.e., recognition of the mRNA codon by the tRNA anti-codon) and peptide-bond formation in the LSU, then moves to the P-site to retain the nascent polypeptide during addition of the next amino-acid, and to the E-site following tRNA deacetylation before leaving the ribosome. During each elongation cycle, both ribosomal subunits play a dynamic role in translocating the mRNA and the tRNA along the ribosome via three nucleotides (see Review [9]). Translation termination occurs when the ribosome encounters a stop codon, which cannot accommodate any of the tRNA anticodon. Instead, the release factor eRF1 is responsible for recognizing stop codons, and induces, with eRF3, the release of the nascent polypeptide from the peptidyl-tRNA located in P-site (see Review [10]).

All of these factors, i.e., eIFs, eEFs, and eTFs, which are part of the translational apparatus are well-characterized key trans-regulators of translation. To exert their functions they are subjected to post-translational modifications, mainly phosphorylation, following activation or inhibition of intra- and extracellular signaling cascades in response to stimuli occurring in physiological or pathological conditions. These modifications impact their activities and subsequently modify the global rate of translation by modulating initiation, elongation and termination rates. Many reviews have described in great details how and by which molecular mechanisms these trans-regulators modulate translational efficiency [5,7,11]. Furthermore, it unexpectedly appears that some of these factors, such as eIF4E, eIF4A, and eIF3, are involved not only in the control of the global rate of protein synthesis but also in the differential translational activity of distinct subsets of mRNAs [12,13,14,15,16].

In addition to these canonical translation factors regulating the three steps of translation, there is an increasing number of trans-regulators that do not belong to the core of the translational machinery, but that interact with it to modulate the translational efficiency of subsets of mRNAs. These additional trans-regulators of translation, including RNA binding proteins (RBPs) [17], internal ribosome entry site (IRES)-trans acting factors (ITAFs) [18], miRNAs [19], or lncRNAs [20] for example act in concert with the canonical translation factors to achieve a finely-tuned translation mainly during the initiation step. Finally, irrespective of their nature and mode of action, these trans-regulators interact with elements of the targeted mRNA harboring either specific structures or sequences that act as cis-regulators. These cis-regulatory sequences are mainly buried within the so-called 5′ untranslated regions (5′UTRs) and 3′ untranslated regions (3′UTRs) of mRNA (see reviews [11,21]). These cis-elements of mRNAs allow direct binding with trans-regulators and consequently contribute to modulate the translational efficiency of the corresponding mRNA. Among them, some are binding sites for RBPs [17] either as specific binding sequences, including cytoplasmic polyadenylation elements (CPEs) [22] and AU-rich elements (AREs) [23], or as secondary structures such as IRES [18], stem-loops [15], and RNA G-quadruplex [24]. In addition, several cis-elements of mRNAs regulate translation without the intervention of trans-regulators, but rather by modulating 80S ribosome formation on the accurate AUG, such as upstream open reading frames (uORFs) [25], 5′-terminal oligopyrimidine tracts (5′-TOP) [26], translation inhibitory elements (TIE) [27], pyrimidine-rich translational elements (PRTE) [28] and cytosine-enriched regulators of translation (CERT) [12]. It has to be noted that chemical modifications of these cis-elements, including m6A, finely modulate their binding with the different trans-regulators (see reviews [21,29,30]).

**Figure 2:**
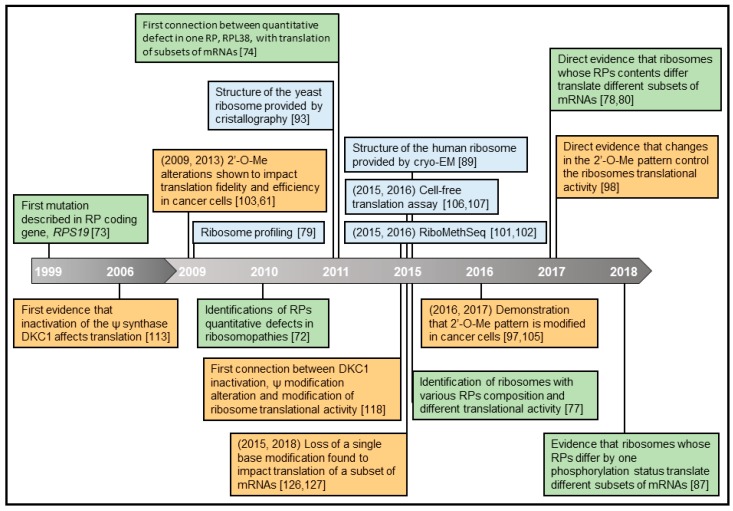
Key manuscripts and associated breakthrough technologies that contributed to demonstrating that a specific ribosome composition determines the subset of mRNAs being translated. First and second decades, 1999–2008 and 2009–2018, are depicted by two grey arrays. Hallmarks colors are as follows: impact of RP defects (green) and rRNA modifications (orange) on ribosome translational activity, as well as breakthrough technologies and ribosome structure provided by recently developed structural technologies (blue).

A notion has been formulated recently that ribosomes themselves could be direct actors in the regulation of translation, in addition to the widely studied mechanisms of translational regulation cited above [31,32,33]. In this review, we will highlight the data and the technological efforts made over the last decade to sustain this theory (Figure 2). We will address the concept of “specialized ribosomes” by presenting recent works focusing on whether heterogeneity in the composition of ribosomes provides them with a preference for translating certain subsets of mRNAs.

## 2. The Ribosome: A Myriad of Factors Regulating Ribosome Abundance

The human ribosome contains 80 ribosomal proteins (RPs) and 4 rRNAs (namely the 28S, the 18S, the 5.8S, and the 5S) organized in two ribonucleo-proteic subunits, the SSU and the LSU. The SSU contains the 18S rRNA and 33 distinct RPs, and the LSU contains the 5S, the 5.8S, the 28S rRNAs and 47 distinct RPs [34]. The ribosome is the effector of translation as it decodes mRNA and synthesizes protein. The SSU contains the mRNA entry and exit sites, the path along which the mRNA progresses and the decoding center, at the heart of which codons are read. The LSU is responsible for peptide bond formation and contains the polypeptide exit tunnel. Importantly, several contact points called inter-subunit bridges, formed of both rRNA and RPs, ensure the assembly of the 80S ribosome and the dynamic coordination between the subunits during translation. rRNAs are essential for the function of the ribosomes in translation. Ribosomes display a ribozyme activity required to catalyze peptide-bond formation and ensure mRNA decoding, as well as protein quality control [35,36,37].

### 2.1. Ribosome Biogenesis

Ribosome biogenesis is a complex process involving more than 150 proteins, and approximately 70 small nucleolar RNAs (snoRNAs) for the synthesis, modification, assembly, and nucleus/cytoplasm shuttling of the 4 rRNAs and the 80 RPs (see reviews [38,39]). There are approximately 10 million ribosomes per mammalian cultured cell, and the rate of ribosomal subunit synthesis has been evaluated at 7500 subunits per minute in the human cancer cell line HeLa [40]. This requires the synthesis of about 300,000 RPs and the transcription of 41 megabases of pre-rRNAs per minute. Indeed, ribosome biogenesis is one of the most energy consuming processes in eukaryotic cells.

The limiting step of ribosome biogenesis is the transcription of rDNA genes by the RNA polymerase I (RNA Pol I), which generates a polycistronic RNA precursor called the 47S pre-rRNA in humans. This precursor is sequentially processed mainly in the nucleoli, generating many rRNA processing intermediates, to form the pre-mature 5.8S, 18S and 28S rRNAs. They are then exported to the cytoplasm where they are submitted to a final maturation to produce the mature 5.8S, 18S and 28S rRNAs [41]. The 5S rRNA is generated by the RNA polymerase III (RNA Pol III), which is also involved in the transcription of tRNAs. RPs are encoded by mRNAs synthesized by the RNA polymerase II (RNA Pol II). Finally, important quality control is exerted all along the process [42].

### 2.2. Regulation of Ribosome Production

Cells tightly regulate their quantity of ribosomes by controlling rDNA transcription. Hence, the amount of ribosomes can vary among different cell types and cell statuses. For instance, the circadian clock timely coordinates the transcription of rDNAs and mRNAs coding for RPs [43] and modulates ribosome assembly [44]. In stem cells, rDNA transcription is differentially regulated, compared to their differentiated progeny, notably through a number of pluripotency-associated factors. Indeed, in stem cells, rDNA transcription is stimulated by highly expressed pluripotent-associated factors that interact with RNA Pol I or with rDNA promoters, while in differentiated cells, rDNA transcription is controlled by scarcely expressed co-transcription factors, such as lineage-specific factors [45]. Compared to normal cells, cancer cells are characterized by an increase in ribosome production [46] mainly through enhanced transcription by RNA Pol I, RNA Pol II and RNA Pol III [47,48,49]. Among the activated oncogenic pathways and inactivated tumor suppressors in tumors, some are recognized as key players of tumorigenesis and have recently been revealed as nodal regulators of ribosome production.

Several oncogenic pathways and oncogenes have been reported as key activators of ribosome biogenesis. The mammalian target of the rapamycin complex (mTOR) is a protein kinase which is activated by several stimuli, including nutrients, hormones and oncogenic signaling pathways. mTOR directly stimulates RNA Pol I and RNA Pol III by interacting with their promoters [50], and thus positively controls the production of the 4 rRNAs and of the repertoire of human tRNAs. In addition to regulate ribosome amount in cells, mTOR regulates translation of mRNAs that contain 5′-TOP elements in their 5′UTR. Since all of the mRNAs coding for RPs contain a 5′-TOP, mTOR positively regulates synthesis of RPs allowing a coordinated synthesis of the different components of the ribosome [51].

Myc has also been described as a regulator of ribosome biogenesis [52]. Myc is known to regulate the transcription of many genes involved in various cellular processes such as cell cycle or apoptosis. Moreover, Myc also directly regulates the efficiency of RNA Pol I transcription [53]. By its direct interaction with the core transcription machinery TFIIIB, Myc also controls transcription by RNA Pol III [54]. Finally, Myc regulates the synthesis of RPs by stimulating the transcription of RNA Pol II [55].

In contrast, some tumor suppressor genes have been identified as inhibitors of ribosome biogenesis. The retinoblastoma protein 1 (RB1) gene was the first gene identified as a tumor suppressor since its mutational inactivation causes human cancer. The first cellular function described for protein pRb, and the most studied, is its pivotal role in the negative control of cell cycle. However, in addition, pRb represses RNA Pol I transcription through binding with the transcription factor UBF [56], and RNA Pol III transcription through its direct interaction with TFIIB [57].

The protein p53, another extensively described protein exhibiting oncosuppressive activity is a multifunctional protein, first known for its activity as a transcriptional regulator. It recently emerged that p53 controls gene expression not only at the level of transcription, but also at the level of protein synthesis through the inhibition of ribosome biogenesis (see reviews [6,58]). Indeed, p53 represses RNA Pol I transcription by interfering with the transcriptional machinery [59] and represses RNA Pol III transcription by interacting with TFIIB [60]. In addition, we have reported that p53 represses the expression of fibrillarin (FBL) which is involved in ribosome biogenesis. p53 binds directly to the FBL gene and inhibits the activity of the FBL promoter. More interestingly, since FBL is a methyltransferase responsible for rRNA 2′-O-ribose methylation, p53 also controls the methylation status of rRNAs in ribosomes [61].

BRCA1 is a tumor suppressor, the main function of which is to maintain genomic integrity via its critical role in DNA damage repair and its involvement in the control of a number of fundamental cellular processes such as cell cycle control, transcription, chromatin structure and apoptosis. However, in addition to its well-characterized functions, BRCA1 was recently identified as a translational regulator. Indeed, our team has shown that BRCA1 regulates protein synthesis through its interaction with PABP1 [62,63]. Furthermore, by its interaction with RNA Pol I machinery and TFIIB machineries, BRCA1 transcriptionally regulates the expression of RNA Pol I and RNA Pol III [64,65].

Interestingly, in addition to the clear evidence exposed above showing that increased ribosome production affects translation, recent data revealed that, according to their status, environment, development, or pathological conditions, cells produce different types of ribosomes that differ in their RP composition (e.g., absence of some RPs, replacement by RP orthologous proteins, post-translational modifications of RPs) and chemical modifications of rRNAs. From these observations the concept of “specialized ribosomes” has emerged [32,66], and could be declined in different physio-pathological conditions with the description of immuno-ribosome [67] or onco-ribosome [68] for examples. Although the concept of “specialized ribosomes” is still in its infancy, solid data have been published from various fields of biology, and convincingly sustain this very provocative view of ribosome contribution to gene expression and cell phenotype. Some of these data are presented and (critically) discussed below.

## 3. Impact of RP Defects on Ribosome Translational Activity

For many years, the ribosome was considered to be a single structure made of 4 rRNAs and 80 RPs, each RP being present as a single copy, thus leading to the notion of a conserved RP stoichiometry. However, while the existence of such a stoichiometric ribosome is irrefutable, it has become increasingly evident that some ribosomes lack certain RPs. Hence, issues such as the abundance of these RP-defective ribosomes, their function, and the subsequent consequences of ribosome heterogeneity on cells have progressively been assessed over the past decade.

### 3.1. From the First Description of Ribosome Heterogeneity to the Concept of “Specialized Ribosomes”

Possibly, one of the first indications that different types of ribosomes existed was derived from the identification of numerous RP gene paralogs in yeast and plants. In 1995, analysis of RPL16 paralog gene expression patterns in Arabidopsis thaliana showed that the two paralogs were mutual exclusively expressed in different organs of the plant [69]. In Saccharomyces cerevisiae, deletion of individual paralogs gave rise to unique phenotypes such as bud size selection [70] or sporulation [71] (see reviews [31,32]). These observations suggested that various types of ribosomes composed of different paralogs encoding RPs were required for specific cellular events.

The evidence that various types of ribosomes may exist in humans came from a cohort of patients displaying genetic diseases collectively called ribosomopathies. Ribosomopathies are caused by haploinsufficiency of genes encoding key factors in ribosome biogenesis or RPs [72]. For example, the Diamond-Blackfan Anemia (DBA) is caused by heterozygous loss-of-function mutations in genes encoding RPs, such as RPS19, RPL5, and RPL11, while the 5q-syndrome is caused by a somatically acquired deletion of chromosome 5q, which leads to haploinsufficiency of RPS14. The first mutation described in RP coding genes was identified in 1999 [73] and corresponds to the first evidence of ribosome heterogeneity in humans. Indeed, the fact that mutation leading to quantitative defects of RPs was observed in the biological context of ribosomopathies strongly suggested that ribosome with altered RP contents occurred.

In 2011, more than one decade after discovery of the first RP coding gene mutation, the first demonstration that a tissue-selective loss of expression of RPs could impact translation of a subset of mRNAs and drastically impact development stemmed from the analysis of the function of RPL38. Using a mouse model characterized by a 50% reduction in Rpl38 gene expression associated with tissue-specific patterning defects, Kondrashov et al. uncovered the important role of RPL38 in translational control by influencing only a subset of mRNAs, rather than affecting global translation. Indeed, a polysome profiling approach enabled this group to isolate and quantify mRNAs present in the polysome fraction and thus actively translated. A subset of 8 mRNAs among the 31 Hox mRNAs, genes crucial for the formation of the mammalian body plan, was found to be regulated in a RPL38-dependent manner [27,74].

Several studies confirmed that RP deficiency impacts translation of mRNA subsets. To identify the mRNAs that were translationally regulated upon depletion of RPS19, a microarray analysis of polysome-bound mRNAs in RPS19-depleted and non-depleted erythroblasts was performed. Upon RPS19 depletion, 130 genes were differentially recruited to polysomes [75]. Using bicistronic constructs that encode two different luciferases, the translation of which is either driven by the cap or by an IRES structure, the authors identified a number of cellular IRES containing mRNA among the translationally deregulated mRNAs [75]. In another study Lee et al. identified by polysome profiling and subsequent mRNA sequencing, a subset of cellular mRNAs, among which a number of stress response transcripts that were selectively sensitive to RPL40 depletion [76].

Studies exploring the effect of RPs deficiency, including, but not limited to those exposed above, provide clues as to RPs deficiency implication in the translational regulation of subsets of mRNAs. However, to support the concept of “specialized ribosomes”, the fact that this translational reprogramming originated from ribosomes of heterogeneous compositions remained to be clearly demonstrated at that time.

Thus, several proteomic-driven analyses explored the whole ribosome protein composition using purified ribosomes under various conditions to identify all RPs differentially expressed between those conditions. Hence, differential stoichiometry among RPs was associated with different translational statuses in murine embryonic stem cells (mESCs) [77]. Indeed, by using the high-coverage tandem mass tag (TMT) mass spectrometry technology, the authors compared the relative quantity of each RP between monosome and polysome fractions. A thorough examination of each RP revealed that some RPs appear to be more abundant in monosomes than in polysomes and vice versa.

Although those previous observations strongly supported the notion of a variation in RP expression then composition, technological improvements are ongoing in order to address directly and more accurately the possibility of a variability in RP stoichiometry. Recently, mass spectrometry was upgraded so as to provide data close to absolute quantification of proteins. This novel technology, called selected reaction monitoring (SRM)-based proteomics, uses the spiking of samples with known amounts of labeled peptides derived from RPs as a standard for absolute quantification. However, such approach being expensive, not all 80 RPs could be spiking for absolute quantification. Nevertheless, using this novel technology, absolute quantification of 15 RPs isolated from polysomes was assessed and 4 RPs were identified as being substoichiometric in mESC ribosomes, namely Rpl10A, Rpl38, Rps7, and Rps25, [78]. Based on this technology, the heterogeneity in ribosome composition was revealed for the first time within a single cell type and a single polysome profile fraction. The authors concomitantly extended their investigation of the potential heterogeneity to all RPs, using the TMT technology. Comparison of the relative quantity of each RP between polysomes and free SSU and LSU identified additional RPs, namely Rpl40, Rps26, and Rpl10, which were more abundant in polysomes compared to others in mESC cells.

To address the impact of ribosome heterogeneity on the translation of subsets of mRNAs, Rpl10A and Rps25 were chosen among the substoichiometric RPs of mESC in this study [78]. Using the CRISPR/Cas9-mediated genome editing, two mESCs lines were generated. One harbored the 3xFAG-Rpl10a allele and thus produced ribosomes containing Flag-Rpl10A (called Flag-Rpl10A-ribosome), while the other contained the Rps25-3xFLAG allele to produce ribosomes containing Flag-Rps25 (called Flag-Rps25-ribosome). An adapted ribosome profiling approach, based on tagged-ribosome immunoprecipitation and subsequent ribosome profiling was tested. Ribosome profiling is an approach based on deep sequencing of ribosome-protected mRNA fragments that enables the analysis of translated sequences at sub-codon resolution [79]. This modified ribosome profiling technology enabled the group to identify mRNAs that were specifically bound to Flag-Rpl10A-ribosomes or Flag-Rps25-ribosomes. In parallel, conventional ribosome profiling provided the identification of mRNAs protected by total ribosome content in mESCs. By comparing sets of mRNAs bound to all ribosomes with those bound to Flag-Rpl10A-ribosome or Flag-Rps25-ribosome, the authors concluded that Rpl10A-containing ribosomes promote translation of mRNAs encoding extracellular matrix proteins, while Rps25-containing ribosomes coordinate the translation of mRNAs encoding proteins involved in vitamin B12 signaling.

This study therefore demonstrated in 2017 that heterogeneity in ribosome protein composition provides a given ribosome with a specificity for translating subsets of mRNAs, and thus strengthened the concept of “specialized ribosome”.

Finally, a study published the same year reinforced this notion by demonstrating that ribosomes depleted in a given RP exhibit different translational activities compared to non-depleted ribosomes. Indeed, Ferretti et al. used a yeast system to assess whether RPS26, which is frequently mutated in DBA, influences the repertoire of translated mRNAs [80]. A TAP tag-based purification method was used in yeast cells in which Rps3-TAP production was induced when Rps26 and Rps3 production was repressed. This technology enabled the isolation of Rps26-depleted ribosomes from Rps26-containing ribosomes in the same cells. Since each total ribosome pool encountered the same mRNAs, sequencing results of the mRNA-associated with each specific ribosome pool were compared directly. Two distinct subsets of mRNAs were preferentially translated by Rps26-depleted ribosomes and Rps26-containing ribosomes. Rps26-depleted ribosomes preferentially translated mRNAs implicated in stress response, while Rps26-containing ribosomes preferentially translated mRNAs implicated in translation. In addition, exposure of yeast to stress lead to the formation of Rps26-depleted ribosomes and to an increase in the translation of their target mRNAs. Further analysis of the mRNAs preferentially translated by each pool of ribosomes revealed different characteristics. Indeed, mRNAs translated by Rps26-depleted ribosomes lacked conservation of all Kozak sequence elements, while mRNAs translated by Rps26-containing ribosomes present full Kozak consensus. The tag-based purification method used enabled the authors to manipulate each pool of ribosomes separately, and to demonstrate that specialized ribosomes were produced and that they selectively translated mRNAs with specific features. This work thus demonstrated at the molecular level that ribosomes, which differ in their RP composition, are associated with different mRNAs in polysomes.

### 3.2. Post-Translational Modifications of RP, an Additional Layer of Heterogeneity

Although the initial proteomic studies to identify post-translational modifications of RPs date back to the years 2000 [81,82], the precise number of RPs that are post-translationally modified is still not fully elucidated and the role of these modifications in ribosome function remains to be understood [83]. However, it has to be said that most of the RPs bear post-translational modifications such as acetylation, methylation, ubiquitination, phosphorylation, and O-GlcNAcylation, and that over 2,500 post-translational modifications of human RPs have been listed [84].

The archetypal RP modification is undoubtedly RPS6 phosphorylation, which was associated with various physiological and pathological cellular contexts. Yet, despite a plethora of studies, whether ribosomes with phosphorylated RPS6 exhibit functions different from those harboring the unphosphorylated form remains debatable. Only one study suggests that RPS6 phosphorylation stimulates translation of a specific class of mRNAs containing a 5′-TOP sequence, in response to mTOR signaling, however it remains to be demonstrated that such alteration of translation results from phosphorylated RPS6-containing ribosome [85].

Mutations in the leucine-rich repeat kinase 2 (LRRK2), which lead to Parkinson’s disease (PD), is another example. Martin et al. identified RPS15 as a substrate of the LRRK2 kinase [86]. They showed that the activated mutant LRRK2 stimulates both cap-dependent and cap-independent mRNA translation in Drosophila PD models and PD patients. Blocking phosphorylation of RPS15 prevents elevated translation and rescues LRRK2 neurotoxicity. Thus alterations of phosphorylation of RPS15 in a physio-pathological context reinforces the notion that post-translational modifications of RPs can impact ribosome activity.

Until recently, no systematic analysis of the effects of RP post-translational modifications on translation was conducted. A new approach, developed recently, called “polysome proteome profiling” (3P) combines sucrose gradient fractionation with stable isotope labeling using amino acids in cell culture (SILAC)-based quantification [87]. This technique provides proteomic maps along polysome profiles. Interestingly, combining 3P and phosphoproteomics revealed differences in the state of phosphorylation of RPs along the polysome profile. In particular, RPL12 phosphorylation on serine 38 (pS38) was highly abundant in both 60S and 80S fractions but not in the polysome fraction, suggesting that pS38-RPL12 may regulate translation. In particular, phosphorylation of RPL12 does not seem to impact global protein synthesis, but rather regulates the translation of subsets of mRNAs coding proteins regulating mitosis.

Altogether, these studies demonstrate that post-translational modifications of RPs can regulate translation and extend previous findings that heterogeneous ribosomes preferentially translate specific subsets of mRNAs.

## 4. Impact of rRNA Modifications on Ribosome-Mediated Regulation of Translation

rRNAs are central to translation, by directly supporting many of the key molecular interactions driving this process. This includes mRNA decoding [9], peptide-bond formation [88], and inter-subunit bridges [34,89]. Since these RNAs are subjected to intense post-transcriptional modifications, scientists wondered whether rRNA modifications modulate the functions of the ribosome and, as a consequence, impact protein synthesis. Indeed, rRNAs are extensively modified with at least 10 base methylations and acetylations, 95 pseudouridylations (ψ) and over 106 2′-O-ribose-methylations (2′-O-Me) being reported in human rRNAs, and this list is continuously growing [90,91,92].

Recently developed structural technologies, such as X-ray crystallography and cryo-EM, provided new insight into the specific organization of eukaryotic and human ribosomes [89,93]. For instance, crystal structures of the bacterial ribosome have enabled the modeling of rRNA modifications [94]. More recently, cryo-EM structures of human ribosomes clearly established the involvement of rRNA modifications in structuring the ribosome [95,96]. These modifications interact with mRNAs and tRNAs, and are located within the interior of the rRNA thus stabilizing rRNA structures. Strikingly, modified sites are concentrated within the most important functional domains of rRNA, such as the peptidyl transferase center, the decoding center, or rRNA helixes that bridge the two subunits [90].

Hence, a few years ago, scientists intensified their investigations into whether rRNA modifications promote ribosome heterogeneity and impact ribosome activity, including ribosome-mediated regulation of translation as shown for RPs (Figure 2).

### 4.1. 2′-O Ribose-Methylation: A Novel Source of Specialized Ribosomes

Among the different types of chemical modifications, 2′-O-ribose-methylation (2′-O-Me) is the most abundant, with 106 sites mapped in human rRNA [97,98]. 2′-O-Me is carried out by the rRNA methylation complex that contains the methyl-transferase FBL in association with scaffolding proteins, NOP56 and NOP58, the RNA binding protein NHP2L1 (or 15.5kDa protein) and a single snoRNA from the C/D box snoRNA family, which define the ribose to be modified [90,99].

All ribosomes were first described as being methylated at each 2′-O-Me site, leading to the concept that 2′-O-Me was a constitutive rRNA modification in healthy proliferating eukaryotic cells [100,101,102]. We reported for the first time that, in humans, rRNA 2′-O-Me can be altered, however not at all but at some given sites. Alteration of rRNA 2′-O-Me was associated with modulation of mRNA translation during mammary tumorigenesis [61,103]. In breast cancer, FBL over-expression alters rRNA 2′-O-Me patterns, triggers changes in translational fidelity and promotes translation of subsets of mRNAs involved in tumorigenesis and cell survival, such as IGF1R and c-Myc [61]. These early studies were performed with a RT-qPCR based approach that only provided a relative level of methylation alteration. In 2015, a novel OMICs approach was published named RiboMethSeq, which is dedicated to analyzing 2′-O-Me rRNA using a RNA-seq based approach and corresponding to an absolute measure of methylation frequency [91,101,102,104]. RiboMethSeq studies showed that 2′-O-Me can be significantly modified at some given sites in cells upon FBL knockdown, demonstrating that cells tolerate the production of ribosomes with significant modifications in 2′-O-Me patterns [98,105]. These studies firmly established the potential plasticity of rRNA 2′-O-Me, and led to studies focusing on whether 2′-O-Me altered ribosomes carried different translational properties.

To that end, the hybrid in vitro translation technique also called cell-free translation assay [106,107], which is composed of reticulocyte lysates, in which rabbit ribosomes are replaced by ribosomes isolated from cells of interest, provides an optimal model for analyzing intrinsic functional properties of selected ribosome populations. Using purified ribosomes with different rRNA methylation profiles induced by FBL knockdown in cell-free translation assays, it was demonstrated that IRES-dependent translation initiation from IGF1R and some viral RNAs elements, was directly affected by rRNA methylation [98]. Interestingly, several of these elements are prone to IRES-dependent translation through direct interaction with rRNA, although the contribution of 2′-O-Me in such direct interactions remains to be established.

It is well-known that 2′-O-Me is more densely localized within the functional domains of rRNA, notably the decoding center, the peptidyl transferase center (PTC) and intersubunit bridges [100], suggesting they may play a role in the structuring of these domains. The localization of the nucleotides displaying an altered 2′-O-Me pattern upon FBL knockdown was analyzed in the 3D structure of the ribosome [98]. Strikingly, several affected 2′-O-Me sites are located near sites involved in translational processes, including the A and P sites, the intersubunit bridges and the peptide exit tunnel. Yet none of the sites localized close to the PTC was affected, suggesting that this region might be protected from variation in 2′-O-Me.

Thus, 2′-O-Me represents a source of ribosome population heterogeneity observed in different physio-pathological contexts, that preferentially affects several functional domains of ribosomes and impacts ribosomal activity (see review [91]).

### 4.2. Pseudouridylation

Another major rRNA chemical modification consists in the isomerization of uridine into pseudouridine (ψ) [92]. ψ is the 5-ribosyl isomer of uridine. It is derived from the 180° base rotation of uracil which is then attached to the 1′-carbon (C1’) of the ribose via a carbon-carbon instead of a nitrogen-carbon glycosidic bond [108,109]. ψ occurs in all species and in many classes of RNA including tRNA, rRNA and small nuclear RNA (snRNA). In rRNA, ψs account for about the 1.4% of all bases, with a total of 95 predicted ψs in human 28S, 18S, 5.8S, and 5S rRNAs [110]. rRNA pseudouridylation is carried out by RNA-guided enzymatic complexes called H/ACA box RNPs, each consisting of one H/ACA snoRNA and four core proteins, namely GAR1, NHP2, NOP10 and the pseudouridine synthase dyskerin (DKC1) [111].

DKC1 is a ψ synthase that converts uridine residues into ψ in rRNA and snRNA. The role of DKC1 in rRNA pseudouridylation and its impact on ribosome function have been mainly approached using DKC1 functional inactivation models. X-linked dyskeratosis congenita (X-DC) is indeed a rare multisystemic inherited syndrome caused by mutations of the DKC1 gene, characterized by failure of proliferating tissues and associated with an increased risk of developing tumors [112].

In 2006, a study unveiled that functional inactivation of DKC1 modifies translation, quantitatively and qualitatively. Indeed, in cells derived from DKC1-depleted mice or X-DC patients bearing DKC1 mutation, translation of mRNAs containing IRES elements, including those encoding the tumor suppressor p27 and the anti-apoptotic factors Bcl-xL and X-linked inhibitor of apoptosis protein (XIAP) was impaired [113]. Several studies then supported the notion that DKC1 inactivation, and thus rRNA pseudouridylation defects, are crucial for translational control and cell fate [114,115,116,117].

A thorough examination of the molecular mechanisms through which translation is deregulated upon DKC1 inactivation was conducted at the level of ribosomes. As such, ribosomes from DKC1 depleted human cells were purified in order to study whether DKC1 depletion impacts ribosome composition and function. By means of cell-free translation assay, the alterations in IRES-mediated translation, found in cells lacking DKC1, could definitely be ascribed to an intrinsic ribosomal defect [118].

Analysis at the atomic level of ribosome structures indicate that these modifications participate in rRNA folding and are often located in the vicinity of sites involved in ribosome interaction with tRNA and mRNA [90,95].

Thus, from these data obtained through recent technologies, we can propose that, similarly to 2′-O-Me, pseudouridylation modulates the ribosome translational activity [113,116,117,118,119]. However, technical limitations have so far impeded the study of ribosome heterogeneity. While RiboMethSeq mapping of 2′-O-methylated rRNA residues revealed some incomplete modifications at several sites [97], implying that ribosome population is heterogeneous, the equivalent high-throughput approach for ψ identification and quantification [120,121,122] remains less efficient when applied to rRNA [92].

### 4.3. Methylation of Bases

Finally, a few rRNA bases are also modified. In yeast, there are 12 modified bases on ribosomes. These modifications occur through the addition of one, or sometimes two methyl groups onto specific atoms, including at positions 1, 6, and 7 on the purine rings and positions 1, 3, and 5 on the pyrimidine rings [90]. Specifically, the ribosome SSU of budding yeast contains three methylated residues in the small subunit (m7G1575, m62A1781, and m62A1782), and six methylated residues in the large subunit (m1A645 and m1A2142, m5C2278 and m5C2870, and m3U2634 and m3U2843). In the SSU, the pyrimidine ring can also be aminocarboxypropylated at position 3 and acetylated at position 4 (ac4C1280 and ac4C1773). Finally, the 18S rRNA contains a complex N1-methyl-N3-aminocarboxypropylpseudouridine hypermodification (m1acp3cψ1191). Most of these base modifications are conserved in humans, while it is suggested that additional modifications are specific to human ribosomes [95]. The enzymes responsible for introducing base modifications in human ribosomes have been identified [90]. However, it remains unclear whether rRNA bases can be partially modified.

Base modifications have been linked to development and disease. For example, point mutation of the EMG1 gene encoding the enzyme responsible for the m1acp3cψ1191 modification, causes Bowen–Conradi syndrome [123]. Deletion of WBSCR22 and WBSCR20/NSUN5 genes implicated in m5C2278 and m7G1575 modifications, respectively, cause the Williams–Beuren syndrome [124]. However, it is not clear whether pathogenicity could be attributed to altered rRNA base modifications and altered ribosome quantity or activity. Nevertheless, base methylations have been implicated in translation regulation. In yeast, cells expressing inactive DIM1, the enzyme responsible for base methylation of A1781 and A1782 residues, displayed a decreased translation in vitro [125]. In yeast depleted in NSUN5 that is responsible for m7G1575 modification, translation fidelity was affected. In addition, polysome profiling combined with microarray analysis showed that loss of m7G1575 modification modulates the translation of a subset of mRNAs [126].

In yeast abolition of a single base modification, i.e., m1A645 located on the LSU, leads to a change in the quantity of a subset of 18 proteins [127]. Comparison of the protein abundance with mRNA levels of each protein candidate showed that mRNA content did not correlate with protein content for some of the candidates. This suggests that m1A645 modification may impact the translation of only a subset of mRNAs, in particular those encoding key metabolic enzymes.

Thus base modification also contributes to modulating ribosomal activity, though whether these modifications are altered in physiological or pathological contexts remains to be studied in details.

## 5. Conclusions

**Figure 3:**
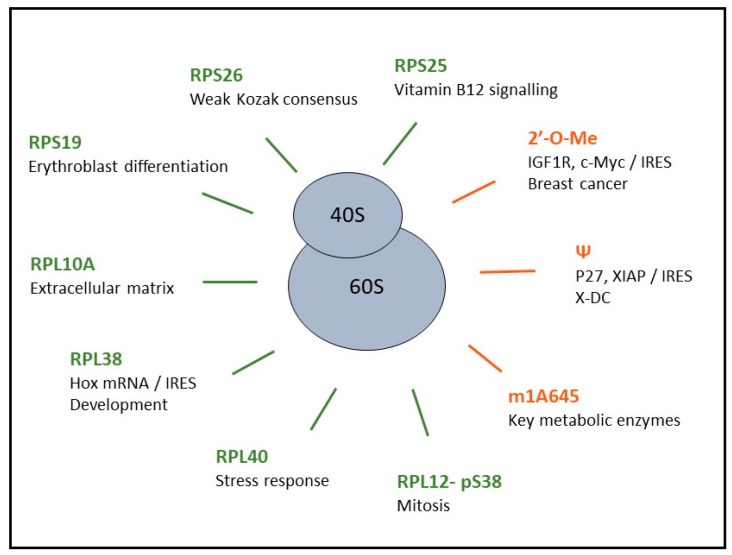
Heterogeneity in ribosome composition impacts translation of various mRNAs subsets. Examples of alteration in the ribosome composition (RP defect or post-translational modification, or rRNA modification) and associated features of preferentially translated mRNAs. Hallmarks colors are as follows: ribosomal protein (RP) defects (green) and rRNA modifications (orange).

For several decades, the ribosome was mainly considered to play a constitutive role rather than a regulatory function in translation. This view was challenged by results showing that cells can produce different types of ribosomes, characterized by variable RP contents and rRNA chemical modifications. These findings led to formulating the concept of “specialized ribosomes” [31,32], which proposes that ribosomes with different biochemical compositions are produced and that each one of these ribosome populations carries different translational abilities (Figure 3). After several decades of accumulating evidence, direct demonstration that specialized ribosomes do exist is only starting to appear, and has led to exciting new perspectives in this field of research [78,98].

An additional source of heterogeneous population of ribosomes may arise from the huge diversity of rDNA variants recently reported [128]. Indeed, in the human genome, the number of rDNA copies ranges from 200 to 400 per cell. Interestingly, rDNA genes contain hundreds of nucleotide variants, most of them being concentrated in the rRNA sequence. Some nucleotide variations are present in translationally active ribosomes and exhibit tissue-specific expression. These findings suggest that rRNA alleles may shape different subpopulations of ribosomes and subsequently influence gene expression.

Interestingly, the concept of “specialized ribosomes” was challenged by the ribosome abundance concept [84,129,130]. Indeed, a recent study argued that reduced ribosome abundance, and not the ribosome composition, selectively impairs translation of a subset of mRNAs [131]. In cells depleted in DBA-associated RPs, including RPS19 or RPL5, a reduced content of ribosomes was observed and ribosome profiling identified a common set of mRNAs whose translation was impaired. Thus, it is most likely that the specialized ribosome concept does not exclude the ribosome abundance model. Indeed, it is conceivable that a population of ribosomes can be characterized by a global modification in ribosome abundance and the concomitant presence of specialized ribosomes, suggesting that the two concepts may explain together selective impairment of translation.

The existence of ribosome heterogeneity adds numerous layers of subtlety when speaking about translational regulation. However, deciphering the fine-tuning of ribosome-mediated translational regulation requires in-depth scrutiny of ribosomes. The next challenges will be to identify and quantify each rRNA post-transcriptional modification, and to quantify each RP in one single cell and then in one single ribosome. Although innovative quantitative mass spectrometry approaches measuring the absolute abundance of RPs have proven their pertinence potential [78], further improvement of mass spectrometry technologies to provide absolute quantification of each of the 80 RPs composing the ribosome will provide a better and more precise view of the cellular diversity of the riboproteome. An exciting alternative to analyze ribosome composition relies on a modified mass spectrometry protocol, which does not require digestion of protein and which enables high-resolution analysis of native protein complexes as large as 9 MDa [132]. Single-molecule sequencing approaches, such as nanopore sequencing [133], are also highly promising as they enable the direct sequencing of a single RNA molecule without requiring its conversion into cDNA. This technology, currently applied to DNA and cDNA [134], could be optimized to detect RNA modifications in the near future [135]. To decipher how rRNA modifications and RP stoichiometry finely control translation by ribosomes, both in a physiological and pathological context it will be necessary to analyze the translating ribosome as a whole, by simultaneously considering mRNA, tRNA and ribosome status. Consistently, a genome wide Ribo-tRNA-seq assay was recently set up to analyze, at a large scale, the identity and the abundance of ribosome-embedded tRNAs and their modifications [136].

Translation deregulation in human disease is a rapidly developing field of research, and some molecules are already in use to treat human diseases, including neurodevelopmental syndromes and cancer [129]. In this emerging context, targeting ribosomes, either their production or their function, represents a very promising strategy to engineer cancer-related drugs with high specificity toward cancer cells. Recent structural analysis of prokaryotic and eukaryotic ribosomes demonstrated that molecules, such as antibiotics and chemical inhibitors of translation, can bind to the ribosome. By their heterogeneity in their RP content and rRNA modifications, specialized ribosomes could present a panel of subtle differences in their structure. In a pathological context such as cancer, these differences could be targeted and may thus represent new therapeutic candidates in the near future [137,138,139].

## Abbreviations

2′-O-Me	2′-O-ribose-methylation
5′TOP	5′-terminal oligopyrimidine tract
ARE	AU-rich element
CERT	cytosine-enriched regulator of translation
CPE	cytoplasmic polyadenylation element
DBA	Diamond-Blackfan Anemia
DKC1	dyskerin
eEF	eukaryotic elongation factor
eIF	eukaryotic initiation factor
eTF	eukaryotic termination factor
FBL	fibrillarin
IRES	internal ribosome entry site
ITAF	IRES- trans acting factor
LRRK2	leucine-rich repeat kinase 2
LSU	60S large subunit of ribosome
mESC	murine embryonic stem cell
mTOR	mammalian target of rapamycin complex
PABP1	poly(A)-binding protein 1
PD	Parkinson’s disease
pRB	Retinoblastoma protein
PRTE	pyrimidine-rich translational element
PTC	peptidyl transferase center
ψ	pseudouridylation
RBP	RNA binding protein
RNA Pol	RNA polymerase
RP	ribosomal protein
snoRNA	small nucleolar RNA
SRM	selected reaction monitoring
SSU	40S small subunit of ribosome
TIE	translation inhibitory element
TMT	tandem mass tag
uORF	upstream open reading frame
UTR	untranslated region
X-DC	X-linked dyskeratosis congenital
XIAP	X-linked inhibitor of apoptosis protein
